# Physicochemical Principles of Adhesion Mechanisms in the Brain

**DOI:** 10.3390/ijms24065070

**Published:** 2023-03-07

**Authors:** Katarzyna Stachowicz

**Affiliations:** Department of Neurobiology, Maj Institute of Pharmacology, Polish Academy of Sciences, Smętna 12, 31-343 Kraków, Poland; stachow@if-pan.krakow.pl

**Keywords:** adhesion, CAMs, cell adhesion molecules, surface tension, forces, brain

## Abstract

The brain functions through neuronal circuits and networks that are synaptically connected. This type of connection can exist due to physical forces that interact to stabilize local contacts in the brain. Adhesion is a fundamental physical phenomenon that allows different layers, phases, and tissues to connect. Similarly, synaptic connections are stabilized by specialized adhesion proteins. This review discusses the basic physical and chemical properties of adhesion. Cell adhesion molecules (CAMs) such as cadherins, integrins, selectins, and immunoglobulin family of cell adhesion molecules (IgSF) will be discussed, and their role in physiological and pathological brain function. Finally, the role of CAMs at the synapse will be described. In addition, methods for studying adhesion in the brain will be presented.

## 1. Adhesion as a Physicochemical Phenomenon

**Figure 1:**
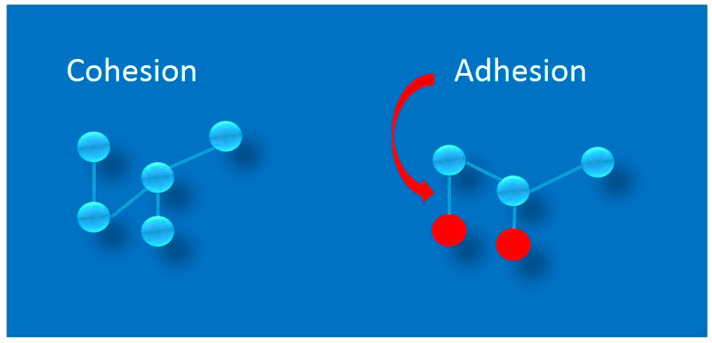
Schematic diagram showing the physicochemical principles of adhesion and cohesion, indicating the differences between these phenomena. Blue spheres represent similar or identical molecules, e.g., water, while red spheres represent different molecules, e.g., nitrogen.

In the cell-adhesion–is a binding of a cell to another cell, or a cell to a surface via specific cell adhesion molecules (CAMs) [1]. In physics, adhesion is a phenomenon involving the joining together of different layers, but also phases or compounds [2]. However, in both cases, the right conditions must be met for there to be forces of attraction between the adhering particles, which is related to the appearance of the Van der Waals-force type of interactions [3]. Adhesion, should be distinguished from the phenomenon of cohesion. Cohesion is the joining of similar or identical molecules, such as water (water molecules attract each other to form water droplets), while adhesion refers to the joining of different molecules [2]. Adhesion is thus the result of intermolecular interactions between different surfaces [2]. A measure of adhesion is the work that must be done to separate the surfaces that are adhering. In the process of joining surfaces, adhesion can occur or chemical bonds can be formed. These processes are very fluid and difficult to grasp, because atoms in particles strive for an energetically optimal distribution of electrons around nuclei, so as soon as there are favorable conditions for the reaction to occur, chemical bonds will form. Adhesion is defined as a reversible thermodynamic process using surface tension at the interface between layers. A thermodynamic process is a chain of physical phenomena linked to the exchange of work and heat with the environment [2]. It has been experimentally proven that adhesion is directly proportional to the actual contact perimeter, but is not proportional to the contact area [4]. This means only that on a given surface there may be points of stronger or weaker adhesion, while the adhesion of the entire surface will be the resultant of all these points of contact. Adhesion and cohesion are shown in Figure 1.

When considering the physical principles of adhesion, it should be taken into account that the adhesion of cells or cell elements to the surrounding environment induces the generation of intracellular contractile forces, the amplitude of which must adapt to the mechanical properties of the environment [5]. This process is dynamic in nature. Interaction with neighboring cells by physical forces enhances cell adhesion and influences cell contractility [5], thus sensing physical signals is transduced into biochemical signaling, affecting the cell’s response (differentiation, cell growth, cell death, cell signaling) [5]. Useful for determining physical parameters can be: *shear stress* (τ) is the component of the stress coaxial with the cross-section of the material [6]. It results from the shear force, a component of the force vector parallel to the material cross section. *Membrane fluidity* refers to the viscosity of the lipid layer of a cell membrane or synthetic lipid membrane [7]. *Young’s modulus* (E, tensile or compressive modulus), is a mechanical property that measures the tensile or compressive stiffness of a solid material when a force is applied longitudinally. It quantifies the relationship between tensile/compression stress σ (force per unit area) and axial strain ε (proportional strain) [8]. Physical properties, including external forces, topography, and elastic properties affect cell adhesion and migration [5]. E.g., by changing membrane elasticity through ATP-driven activity, decomposition of the F-actin network, remodeling of the acto-myosin cytoskeleton, or acidosis-they can change the rate of adhesion binding, strength or become mobile, forming protrusions affecting cell behavior [7,9]. The above parameters can be precisely measured, through techniques described in the last paragraph of the review.

Cell adhesion plays a key function during the formation of tissues, organs, or the entire organism, as well as in the demonstration of higher-order functions of living organisms such as neuronal communication [1]. However, adhesion parameters are influenced by factors such as the number of adhesion molecules on the cell surface or matrix, the distance the binding domain of the adhesion receptor protrudes from the cell membrane, the lateral mobility of receptors, receptor dimerization and the clustering of receptors on membrane domains. The enhancement of adhesion within the cell membrane occurs, among other things, through the binding of adhesion domains to components of the cytoskeleton, enabling the clustering of receptors that enhance adhesion, thus promoting cell spreading or migration [10]. Specific kind of adhesion is contact between synapses in the central nervous system (CNS) during neurotransmission, a phenomenon provided by CAMs. Synapses are regulatory points in neuronal networks and are characterized by multi-protein complexes distributed on closely adjacent pre- and postsynaptic terminals [11]. CAMs through cell-to-cell connections dynamically regulate the formation of new synapses or modulate the function of existing synapses through protein-protein interaction signaling cascades [11]. The following chapters were devoted to analyzing the current knowledge about CAMs.

## 2. Families of Cell Adhesion Molecules Present in the Brain

**Figure 2:**
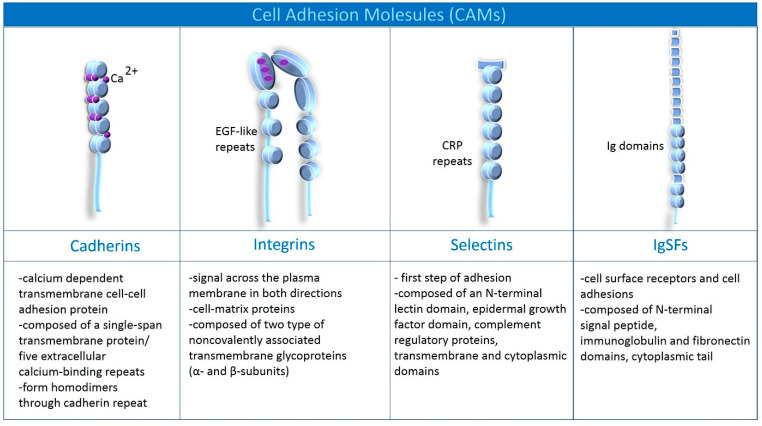
Classification and composition of cell adhesion molecules (CAMs) [14,15,16,17,18].

There are four leading families of CAMs in the brain. They are diverse in structure, calcium dependence, and the signaling they promote [12]. However, the common phenomenon they induce is adhesion in the brain (Adhesion applies to the entire organism’s cells; however, we will focus only on CAMs and mechanisms documented in the brain) [12]. Among CAMs are cadherins, integrins, selectins, and immunoglobulin superfamily of cell adhesion molecules (IgSF) [12]. Cadherins are classified into classical cadherins (N-cadherins found in neurons, E-cadherins expressed in endothelium and neurons) and protocadherins (expressed in neurons: clustered and unclustered) [13]. As for integrins, they are divided into seven subgroups: α5β1 (expressed in B and T cells, astrocytes and endothelium), α1β1 (expressed in astrocytes and endothelium), α6β1 (expressed in T cells, microglia, oligodendroglium, endothelium), αvβ3 (expressed in B and T cells, monocytes, endothelium, microglia, oligodendroglial), αLβ2 (expressed in leukocytes, macrophages, T cells, microglia), αMβ2 (expressed in B cells, NK cells, macrophages, microglia), αXβ2 (expressed in dendritic cells, B cells, macrophages, microglia). Selectins have been divided into E-selectins (endothelium, activated astrocytes), P-selectins (endothelium, platelets) and L-selectins (leukocytes). Among the IgSF can be distinguished ICAM-I (in endothelium, astrocytes, microglia), NCAM (specific for neurons), VCAM-I (in endothelium and monocytes), PECAM-I (in endothelium and leukocytes), and LICAM (in neurons) [13]. The main classification of CAMs is shown in Figure 2.

**The cadherin** superfamily represents calcium-dependent transmembrane cell-cell adhesion proteins [14,15,17]. Calcium dependence means they require Ca^2+^ to function and are protected by Ca^2+^ from cleavage by proteases [19]. Cadherins are composed of a highly conserved cytoplasmic domain (that allows for specific interactions with intracellular binding partners), a single-span transmembrane protein, and five extracellular calcium-binding repeats (cadherin motifs/EC domain) [15,18]. See Figure 2 for details. Classical cadherins are divided into subclasses based on immunological specificity epithelial (E-), placental (P-), and neural (N-) [19,20]. However, the human body has one hundred and fifteen known members of the cadherin superfamily, divided based on structure and function into 12 families [20]. The classification is not closed herewith; in the literature, we find a different number of cadherin families [20]. Binding partners for cadherins, among others, are α-catenin, β-catenin, placoblobin, p120-catenin, presenilin, Shc, N-methyl-D-aspartate receptors (NMDARs), alpha-amino-3-hydroxy-5-methylisoxazole-4-propionic acid receptors (AMPARs; GluR2), postsynaptic density proteins-exchange factors (PDZ-GEF) [20]. Those willing to explore the long list of partners for cadherin are referred to the review of Hirano and Takeichi [20]. According to current knowledge, a cadherin monomer interacts with another cadherin monomer on the plasma membrane, forming *cis*- or *trans*-dimers [20]. For trans-dimerization, the extracellular domains form an X-shaped trans-dimer near EC1-EC2 (cadherin motifs), and sequentially the tryptophan 2 (Trp 2) residue in EC1 cadherin aligns with the hydrophobic pocket of the other molecule [20]. The interaction above occurs through a reciprocal mechanism between monomers called “strand exchange” [20]. The cytoplasmic domain is essential for producing cell-to-cell solid adhesion involving catenins [20]. This mechanism forms neural networks, including cell recognition and signal transduction through cytoplasmic domains [20]. The expression of cadherins begins during neurulation, to be expressed as puncta that correspond to synapses or perisynaptic areas in the adult brain [20].

**Integrins** link the extracellular matrix to the cytoskeleton and are involved in the transfer of the signal across the plasma membrane in both directions [14,15,21]. They modulate ion channels (Ca^2+^ and K^+^), intracellular Ca^2+^ concentration, protein kinase activity, cell contractile properties, and growth factor receptors [22]. Integrins are heterodimeric transmembrane glycoproteins composed of two non-covalently associated subunits: α and β [16,22]. They look like two legs anchored in the cell membrane and connected by their heads in the extracellular space (see Figure 2) [16]. Both subunits are composed of a cytoplasmic domain, a single membrane-spanning helix, and an ectodomain with a ligand-binding surface [14,15,21]. Ligand binding depends on cations, such as Mg^2+^, Ca^2+^, and Mn^2+^, that bind on a surface between the two subunits [21]. This part of integrins is known as the “metal-ion-dependent adhesion site” (MIDAS) [21]. For a detailed description of the complex structure of integrins, see Campbell and Humphries [21]. Integrin subunits are expressed in different regions of the brain, e.g., cerebral cortex, olfactory bulb, hypothalamus, hippocampus, cerebellum, and brainstem [22]. The increased expression has been observed in the axon’s growth cone; in addition, they function in glial cells [22].

**Selectins** are known as a first step adhesion [17,23], and their signal influences the behavior of integrin’s subunits during leucocyte activation [23]. Selectin-dependent rolling allows leukocytes to come into contact with chemokines, activating leukocyte integrins, and enabling arrest and crawling [23]. The compiled mechanism of conformational changes of integrin subunits as a result of activation of selectin signaling has been described in detail in McEver [23]. There are three types of selectins: L-, P-, and E-selectins, distinguished by the number of CRP repeats [23,24]. Each of them is composed of an N-terminal lectin domain (Ca^2+^-dependent), epidermal growth factor (EGF)-like module, complement regulatory proteins, and transmembrane and cytoplasmic domains [17,23]. L-selectins are expressed on the surface of leukocytes, P- on megakaryocytes and endothelial cells, and E- on the surface of bone marrow and skin venous endothelial cells [23].

**The immunoglobulin superfamily of cell adhesion molecules (IgSFs)** is one of the most prominent CAM families, containing surface receptors and cell adhesins [12]. The IgSFs family includes Down Syndrome Cell Adhesion Molecule (DSCAM), Neural Cell Adhesion Molecules (NCAMs), Intracellular Adhesion Molecules (ICAMs), Vascular Cell Adhesion Molecules (VCAMs), Platelet Endothelial Cell Adhesion Molecules (PECAM-1), Endothelial Cell-Selective Adhesion molecule (ESAM) [12,17]. A common feature of the structure of all proteins of this family is the presence of immunoglobulin domains frequently linked by disulfide bonds, transmembrane, and cytoplasmic domains (see Figure 2) [12,17]. IgSFs are distributed throughout the brain, in neurons and glial cells [12].

## 3. CAMs in a Health and a Disease

Adhesion is associated with cancer and the mechanism of cellular infiltration; however, CAMs have a much broader role in the body and are a fundamental functional construct of the brain [12,24,25,26,27,28,29,30,31]; hence this chapter will outline the diseases and cellular processes in which the involvement of CAMs has been documented.

- **Cancer**–One of the CAMs that plays a vital role in cancer is cadherins [18]. It was shown that the E-cadherin-catenin complex is a fundamental part of epithelial monolayers, upholding homeostasis and maintenance [18]. Losing E-cadherins results in cancer progression [18]. One of the mechanisms in that action is the formation of mature junctions that suppress tumor invasion [18]. Furthermore, a loss of E-cadherins releases cellular signals that promote tumor growth and infiltration, e.g., Rho GTPases, PI3K et cetera [18]. The significant role of selectins in tumorigenesis and inflammation has also been demonstrated [16,32]. Selectins, via adhesive mechanisms, protect the organism from bacterial infections and are involved in trafficking cells of the innate immune system, T lymphocytes, and platelets [16]. E-selectin expression increases up to 6 h after inflammatory stimuli, e.g., interleukin-1 [32]. The mechanism selectins utilize is a rolling adhesion through selectin-ligand interactions, described in detail by McEver [23]. Selectins’ role in tumorigenesis has also been established [16,32]. They facilitate the hematogenous dissemination of tumor cells and their arrest in the microvasculature by activating adhesive mechanisms between selectin-expressing host cells and ligands of tumor cells [32].
- **Neurological disorders**–The role of CAMs in neurological disorders is well documented. The connections with depression, schizophrenia, Parkinson’s disease, autism spectrum disorders, Alzheimer’s disease, Down syndrome, epilepsy, and others were found [12,22,25,33,34,35]. When NCAMs are attached to the polysialic acid (PolySia-NCAMs), they play a leading role in schizophrenia as vital regulators of synaptic plasticity, cell migration, axon guidance, and synapse formation [35]. The loss of PolySia-NCAM in NCAM knockout mice resulted in impaired learning, memory, LTP, or LTD [35]. On the other side, increased PolySia-NCAM immunoreactivity was documented in the schizophrenic hippocampus (HC) or prefrontal cortex (PFC) [35]. NCAM isoforms and soluble NCAM fragments were shown as concentrated in the cerebrospinal fluid of schizophrenic patients [35]. The changes in the level of NCAMs or their metabolism correspond with changes in the volume of the left PFC (Brodmann area 46) in schizophrenic patients [35]. Furthermore, PolySia-NCAMs were shown as involved in negative symptoms of schizophrenia [35]. Social affiliative behavior undergoes sCAMs regulation via mechanisms of synapse formation, development, and plastic changes [25].

Intellectual disability also connects with CAMs, documented with DSCAM [12,36,37]. DSCAM plays a role during brain development, and its role in an adult brain is reduced to, e.g., synapse plasticity; however, its massive overexpression is observed in Down syndrome brains [12]. Overexpression of DSCAM connects with morphological and functional changes in vital brain structures to cognition [12]. Decreased spine densities and increased spine volumes were documented in the neocortex and HC of Ts65Dn mouse, which models Down syndrome [38,39]. 

Following discussing mental health conditions, it is vital to mention the involvement of CAMs in depression [12]. The idea was postulated in 2018 by Stachowicz in the context of DSCAM [12]. However, only a few studies on a topic are present. Only recently, Liu et al. [40] documented increased levels of Carcinoembryonic Antigen Related Cell Adhesion Molecule-1 (CEACAM-1) and Neural Cell Adhesion Molecule (NCAM) in plasma of patients with major depressive disorder (MDD).

- **Learning and memory**–involve a vast array of CAMs [12,22,29]; therefore, we will present only selected examples here. Following Wu and Reedy [22], integrins are involved in learning and memory through a few mechanisms. One of the mechanisms is the modulation of long-term potentiation (LTP) [22]. The effect of integrins on LTP was demonstrated by a knockout of the gene encoding α-integrin in Drosophila, resulting in impaired short-term olfactory learning [22,41]. Similar results were documented in mice [22]. Integrins may regulate LTP via modulating N-methyl-D-aspartic acid receptors (NMDARs) or α-amino-3-hydroxy-5-methyl-4-isoxazolepropionic acid receptors (AMPARs) activity [29]. Furthermore, any functional changes associated with integrins result in morphological changes in a synapse structure and function [22]. This is due to the available combination and influence on the scaffolding proteins and actin in spine heads and necks [29]. Cadherins, similarly to integrins, regulate synapse behavior upon NMDA activation [29]. However, their action is required for LTP and spine enlargement, but not for LTD and spine density and morphology, which indicates their participation in synapse plasticity [29]. IgSFs, on the other hand, e.g., NCAMs, regulate synapses via crosslinking to NMDARs and CaMKII via postsynaptic scaffolds [29]. NCAMs may interact with dyneins, enhancing synapse stability, and as states to SynCAMs enlarge during LTD controlling cleft diameter [29]. Our studies found connections between mGluR5, which are postsynaptically localized with DSCAM in a context of spatial learning and depression [42].
- **Circadian clock functions**–are controlled by the Ephrin receptor: EphA4, a CAMs are expressed in the suprachiasmatic nucleus (SCN) [43]. Its documented role in this phenomenon is to regulate the connections of neurons and astrocytes in the SCN [43]. The role of EphA4 in circadian clock functions was documented with EphA4-/- mice use [43]. EphA4-/- mice are characterized by reduced running activity, more extended endogenous periods under constant darkness, and shorter periods under continuous light conditions [43]. In addition, the inactivation of PSA-NCAM (polysialylated neural cell adhesion molecule) was able to extend the length of the circadian clock periods in mice [43,44].
- **Adhesive tissue engineered scaffolds (ATEs)**-have been developed to repair damaged tissues and guide tissue regeneration after injury and degeneration [45]. ATEs have found applications in nerve regeneration, cartilage repair, corneal regeneration, skin regeneration, cardiac tissue repair, bone repair and others [45]. Adhesive scaffolds can be created using various types of biomaterials (hydrogels, assembled microgel spheres, foams, electrospheres) [45]. ATEs provide a three-dimensional (3D) biomimetic and highly biocompatible environment for cell adhesion, growth, proliferation, differentiation, secretion of extracellular matrix proteins, and remodeling or scaffold replacement with regenerated tissue during matrix degradation [45]. This topic was comprehensively described by Chen et al. [45].
  Table 1 presents selected conditions and functions in which CAMs have been confirmed to be involved.

## 4. The Role of CAMs in a Synapse

**Figure 3:**
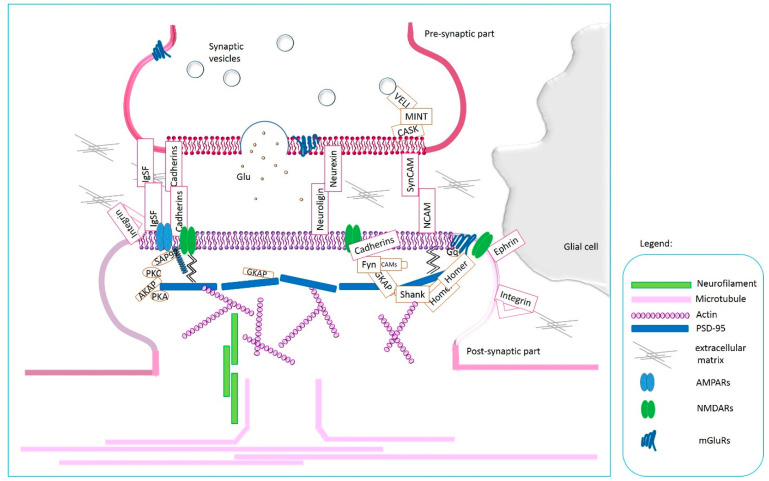
Adhesion at the synapse-a schematic picture showing the ways in which CAMs interact to form a mobile synapse.

CAMs not only act as linkers between cellular elements of the central nervous system, as we may think intuitively but also actively participate in the transmission of signals between neurons and in synaptic plasticity, as has been scientifically documented [12,29,31]. A synapse consists of a presynaptic part, a cleft, and a postsynaptic element. The presynaptic and postsynaptic components communicate by releasing neurotransmitters and gating ion channels of receptors located postsynaptically [47]. CAMs also play their vital role here. Not only do they form connections between synapse elements interacting in a homophilic and heterophilic manner across the synaptic gap, but they are also involved in the transmission of signals through cellular domains and connections to scaffolding and actin proteins [12,29,48]. Synaptically localized adhesion molecules (SAMs) can modify the formation of synapses, modulate the morphology of dendritic spines, influence the function of synaptic receptors and regulate synaptic plasticity [29]. Trans-synaptic signaling in synaptogenesis is mediated via Neurexins/neuroligins, SynCAMs, SALM2, and NGL2; which may induce postsynaptic differentiation via the direct association with postsynaptic density protein 95 (PSD-95) via PDZ binding domain interactions (*results* in vitro and also in vivo). The leading role of PSD-95 in synaptic transmission, synaptic plasticity, and morphological remodeling was described by Stachowicz [47]. EphBs/ephrin-Bs communicate through direct extracellular interactions of EphB2 with NMDARs and AMPARs through the PDZ-binding domain [29]. Subsequently, N-cadherin can bind PSD-95 clusters with cadherin signaling inhibition and interact with AMPARs [29]. On the other hand, presynaptically induced differentiation by neurexins/neuroligins and SynCAM are connected with the CASK/MINT association [29,30]. The functional and structural localization of CAMs at the synapse is shown in Figure 3.

Synapse formation and plasticity are mechanistic processes involving CAMs [29]. The subtype of CAMs present at a synapse can determine what type of synapse will emerge (excitatory or inhibitory) [29]. Moreover, their localization at the synapse determines the specificity of the synapse and how it interacts with its environment and network [29]. Integrins are able to regulate spine function by controlling receptor trafficking in a subunit-specific manner, e.g., α3β1 subunits regulate LTP through NMDARs, while αvβ3 subunits regulate LTP through AMPARs [29]. Cadherins control the formation of spines and synapses in excitatory neurons: stabilization of postsynaptic kainate receptors or AMPARs [29]. Neurexin-neuroligin binding stabilizes dendritic filopodia during synaptogenesis [29]. At mature synapses, neurexin destabilization leads to a reduction in synaptic strength by altering presynaptic release [29]. These are a few examples showing the important role of CAMs in the functionality of synapses and networks. The above determines not only changes in synapses and network communication, but is related to continuous changes in physical forces over time which translates into brain plasticity.

## 5. The Idea of Assessing the Physicochemical Phenomenon of Adhesion in Mental and Cognitive Disorders

Cell adhesion in neurobiology represents the contact between neurons, dendrites, and synapses. The synapse is under constant fluctuation in surface area, surface tension, and continually remodeling to control synaptic plasticity and behavior. Any disturbance in the synapse leads to communication disturbances which may be observed as cognitive or mental changes. Properly functioning synapses must be nearby and have the power to transmit signals; CAMs provide all these conditions. Adhesion mechanisms, as a physicochemical phenomenon, ensure the integrity of cellular structures and connections in the brain. Devastating morphological and structural changes in the brain have been observed in conditions such as schizophrenia, Alzheimer’s disease, or depression [49,50]. The brains of patients with Alzheimer’s disease showed a characteristic reduction in the volume of the PFC, insula, anterior cingulate gyrus, superior temporal gyrus, and gray matter and HC formation [49]. Depressed patients’ brains have morphological changes in cortical and subcortical regions, HC shrinkage, and decreased amygdala volume [50]. Furthermore, morphological changes in neurons, synaptic spines, and dendrites were found in discussed conditions [51,52], and the changes in CAMs were observed [12,22,25,33,34,35]. Adhesive changes follow morphological changes, and eventually, neurotransmission may be dysregulated [12,53]. The regulation above is bilateral [12,53,54]. Cell adhesion bond strengthens the cell with other cells or tissues while supporting the forces involved in cell function [55]. The process is dynamic. Following Evans and Calderwood [55]: “*…a single adhesive bond effectively withstands the force for less time than required for its spontaneous dissociation under thermal activation. Thus, the diversity in the mechanochemistry of adhesive bonds reflects how a mechanical force applied to a bond between a pair of interacting molecules alters the activation energy barriers along kinetic pathways or switches pathways that lead to dissociation…*” In a functional system as complex as the brain, where the energy required for the work of neurons and the formation of new dendritic domains is constantly changing, it is not surprising that there is so much structural and functional diversity among CAMs to ensure the functional integrity of the whole system, e.g., integrins by attaching to the actin cytoskeleton promote strong adhesion and provides conditions for lamellipodium protrusion and locomotion [56]. In migrating cells, the adhesion is temporally and spatially regulated [56]. For details on the mechanical and structural changes of integrin reorganization, see Zhu et al. [56].

## 6. Selected Methods for Evaluating Cell Adhesion

One of the first attempts to study Van der Waals interactions between cell surfaces was described by Nir and Andersen in 1977 [57]. The authors measured the refractive indices of solutions for groups of sugars, phospholipids, and cholesterol at 2500–9000 Å [57]. They documented that the magnitude of Van der Waals interactions between cell surfaces changes according to the sequence: water < phospholipid < cholesterol < protein < sugars [57]. Nowadays, advanced research techniques are used to determine conformational changes, receptor trafficking, receptor crowding or diffusion, protein-protein interactions and others [58,59,60,61,62]. In the field of measuring cell adhesion strength, many techniques have been developed, a few of which will be mentioned here. 

Following Ungai- Salánki et al. [62], qualitative adhesion data can be assessed using **spin and shear flow assays**. This method evaluates the adhesion force between the cell and the substrate [62]. The applied centrifugal force can act in two directions (normal and parallel to the surface to which the cells adhere)-the resultant shear force is parallel to the surface. To detect the elastic modulus of the membrane and internal pressure-**micropipette manipulation tests** can be used [62]. This method measures the deformation of a single cell attached to the tip of a micropipette through a precisely controlled vacuum in the micropipette [62]. The method can measure both quantitatively the adhesion force (by imposing a tension to break the adhesive contact between two opposing surfaces), and an increasing force is used to measure the magnitude of the breaking force [62]. Second, a constant force can be used to measure the duration of adhesion [63]. **Optical tweezers** are used to manipulate cells and organelles [62]. These instruments use a highly focused Gaussian laser beam to trap and manipulate dielectric spherical particles [62]. The force between the bead and the cell is determined by the displacement of the bead from the focus perpendicular to the optical axis [62]. This methodology allows the measurement of membrane tension [62]. In addition, **atomic force and liquid microscopy (AFM)** allows measuring nano-mechanical properties and extracting quantitative parameters of cells, tissues, proteins, nucleic acids [62]. **Single cell force spectroscopy (SCFS)** can be used to measure the adhesion of a single cell to a bio-interface such as tissue, another cell or a ligand-coated surface [62]. **Fluidic force microscopy**-allows measurement of the adhesion strength of single microbial or mammalian cells [62]. And finally, **cell traction force microscopy (CTFM)** allows the study of the force field generated by single cells [62]. For a detailed description of the above methods and their applications, see Ungai-Salanki et al. [62].

**Figure 4:**
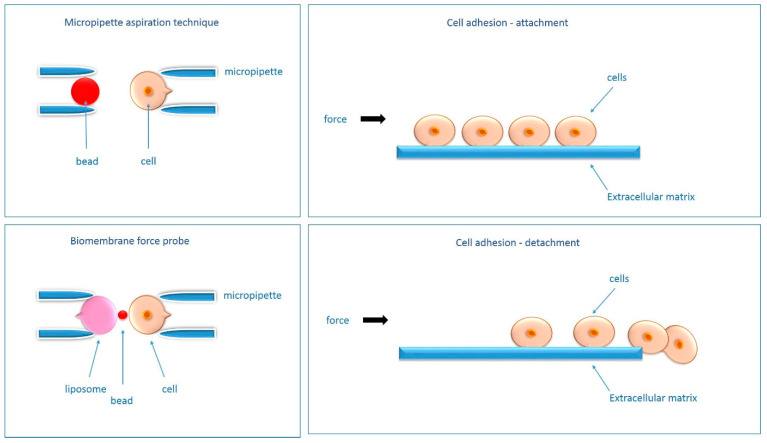
Schematic of selected methods of adhesion detection (micropipette aspiration technique, bio-plasma force probe), including the mechanism of cell attachment and detachment-overview figure. A detailed description of the methods presented can be found in [62,63].

In addition to testing adhesion strength, many methods allow adhesion to be studied through attachment or detachment events [63], which has found applications in biomaterials research, cancer metastasis studies, kinetics studies, drug treatment, signaling pathway studies, and tissue engineering [63]. Among techniques to study **attachment events** should be mentioned: polyacrylamide-traction force microscopy (PA-TFM), micro-patterning, three-dimensional traction force quantification (3D-TFM), wash assay, resonance frequency and microfluidics [63]. Among techniques to study **detachment events** are micropipette aspiration technique, single cell spectroscopy (SCFS), AFM probe force measurement, bio-membrane force probe (BFP), optical tweezers (OT), centrifugation assay, spinning disc, flow chamber and also microfluidics [63]. A description of the aforementioned methodology can be found in Table 2, and a graphical functional approximation of the selected techniques is shown in Figure 4. For more details see [63].

## 7. Conclusive Remarks

Adhesion is a significant physicochemical phenomenon that ensures functional integrity in the central nervous system. During normal brain function, adhesion ensures functional homeostasis of the system. At the same time, during pathological changes, it rearranges the system to ensure, through changes in forces and surface tension, the best possible communication during changing cellular conditions. It is essential to look at this primary physicochemical mechanism and compare the parameters of adhesion in the healthy brain and the pathologically altered one. Correct neuronal communication requires the maintenance of correct physicochemical parameters to reach the crucial areas of brain tissue/neuron/synapses/dendrites/cells with sufficient strength and precision with the neuronal message. Adhesion mechanisms discussed in the article are also essential during ligand/receptor and drug/receptor interactions; however, the topic is not concerned; for details, see Ganesh et al. [33]. What is noteworthy is how many methods exist to measure and study adhesion. From techniques that measure parameters of adhesion forces to methodologies that study cell behavior. The phenomenon of adhesion has a huge potential for application, here we should mention, the study of the behavior of cancer cells, their potential for migration and metastasis. A huge contribution of the adhesion phenomenon can be demonstrated in regenerative medicine with the use of adhesive tissue engineered scaffolds. Interestingly, adhesion mechanisms also allow the study of neurotransmission and fate of synapses.

## Figures and Tables

**Table 1:** Proposed roles for cell adhesion molecules (CAMs) in the brain.

Family of CAMs	Role/Disease	References
Cadherins	-cell recognition, signaling, communication, morphogenesis, angiogenesis, neurotransmission, cancer	[14,18]
Integrins	-cell migration/motility–embryonic development, immune response, tissue repair, cancers -signal transduction cascades–differentiation, proliferation, survival -learning and memory, Alzheimer’s disease, Down’s syndrome, injury and stroke, epilepsy	[22,46]
Selectins	-hematogenous dissemination of tumor cells/cancer	[32]
IgSFs	-cell-cell reorganization, immune system functioning -neuropsychiatric disorders, LTP, synaptic plasticity -changes in the level of DSCAM were found in Down syndrome, Alzheimer’s disease, depression, PTSD -sCAMS as a representing several protein families of CAMs–participate in maintenance, function and elimination of synapses -autism, schizophrenia, social phobia, intellectual disability, attention deficits, bipolar depression, circadian clock functions -changes in a spine density and number, PSD-95 binding, scaffolds regulation	[12,17,25,43]

**Table 2:** Description of the methodology for studying attachment or detachment events during adhesion.

Methodology	Description	Application	Ref.
Cell attachment events			
polyacrylamide-traction force microscopy (PA-TFM)	-technique examines single traction forces-through contact to substrate surface -cells are grown on a polyacrylamide gel with a functionalized cell adhesion ligand and fluorescent beads embedded near the gel surface -as a result of adhesion, the cells generate traction forces that move the beads, and the movement is quantified by tracking the displacement of the fluorescent beads	-real time observation -adaptable to a large variety of cells	[63]
micropatterning	-it relies on basic elastic beam theory -controls micro-patterning approaches involve controlling cellular shape, attachment, spreading	-single cell studies -multi-cellular arrangements in populations of cells -sensitivity and cell response to microenvironment	
three-dimensional traction force quantification (3D-TFM)	-gel-3D cell culture -cells grow inside a gel matrix embedded with fluorescent beads surrounding the cell	-real-time observation -adaptable to a large variety of cells -traction map of tumor cells -the effects of drugs on cells -cell motility -stem cell functions	
wash assay	-static and dynamic culture -after washing–cells that remain adhered to the substrate are analyzed	-for cell count, quantification of DNA content, protein count, antibody binding	
resonance frequency	-changes in resonance frequency are detected at the interaction between cell membrane and substrate	-real-time observation and measurement	
microfluidics	-applies fluid movement during the cell culturing and adhesion -the balance between the adhesive forces generated by the interactions of membrane-bound receptors and their ligands with the dispersive hydrodynamic forces determines cell adhesion	-real-time observation and measurement -cancer cells migration -tumor cell adhesion	
Cell detachment events			
micropipette aspiration technique	-technique detaches an immobilized cell by applying suction force to a portion of the cell surface employed by micropipette suction under observation via a microscope -adhesion strength–minimum force needed to detach a single cell from its substrate	-real-time observation and measurement -mechanical properties of single cells	
single cell spectroscopy (SCFS)	-a microscope is used to observe the cell, while a force is applied to detach the cell using a nanomanipulator, micromanipulator, or micropipette	-real-time observation for short-term adhesion -to study the structures and mechanics of isolated biomolecules, cell nucleus, cytoskeleton	
AFM probe force measurement	-the single cell is immobilized on the AFM cantilever, the live cell is transformed into a probe to measure the adhesion force between cells or the cell matrix	-real-time observation for short-term adhesion -stiffness and adhesion strength against mechanical force	
bio-membrane force probe (BFP)	-using a force transducer (biotinylated erythrocyte) held by a glass micropipette	-real-time observation for short-term adhesion -quantification of a single molecular bond	
optical tweezers (OT)	-using a highly focused laser beam to trap and manipulate particles	-real-time observation for short-term adhesion	
centrifugation assay	-cultures of cells-to assess the strength of adhesion, the number of cells is quantified before and after the load is applied in the centrifuge	-many analysis in parallel	
spinning disc	-takes advantage of the shear stresses generated by the rotating disk device	-high stresses	
flow chamber	-radial or parallel flow chambers	-rea-time cell detachment observation	
